# Pennsylvania Hospital

**Published:** 1849-05

**Authors:** Spencer Sergeant

**Affiliations:** Resident Physician; Pennsylvania Hospital


### PENNSYLVANIA HOSPITAL.—*Surgical Wards.—Service of* DR. PEACE.

### *Cases discharged since March 15th, 1849.*

| | Cured. | By request. | Died. |
|---|---|---|---|
| Abscess, - - - - - - | 0 | 2 | 0 |
| Calculus, - - - - - | 0 | 0 | 1 |
| Contusions, - - - - | 4 | 0 | 0 |
| Chill blains, - - - - - | 1 | 0 | 0 |
| Disease of ankle, (scrofulous,) - | 0 | 0 | 1* |
| Dislocation, - - - - - | 3 | 0 | 0 |
| Erysipelas, - - - - - | 3 | 0 | 0 |
| Fistula Lachrymalis, - - | 1 | 0 | 0 |
| Fractures, simple 6, viz. : | | | |
| Fore-arm, - - - - - | 1 | 0 | 0 |
| Clavicle, - - - - - | 1 | 0 | 0 |
| Patella, - - - - - | 1 | 0 | 0 |
| Thigh, - - - - - | 1 | 0 | 0 |
| Leg, - - - - - - | 2 | 0 | 0 |
| Fractures, compound 4, viz.: | | | |
| Arm, - - - - - - | 1 | 0 | 0 |
| Hand, - - - - - - | 1 | 0 | 0 |
| Leg, - - - - - - | 2 | 0 | 0 |
| Hemorrhoids, - - - - - | 1 | 0 | 0 |

* A delicate child, worn out by a profuse discharge.

| | Cured. | By request. | Died. |
|---|---|---|---|
| Hydrocele, - - - - | 1 | 0 | 0 |
| Inflamed face, - - - - | 1 | 0 | 0 |
| " knee, - - - - | 0 | 0 | 1* |
| " hand, - - - - | 1 | 0 | 0 |
| Onychia, - - - - - | 1 | 0 | 0 |
| Paronychia, - - - - | 2 | 0 | 0 |
| Phymosis, - - - - | 2 | 1 | 0 |
| Sprain, - - - - - - | 1 | 0 | 0 |
| Stricture of urethra, - - - | 1 | 0 | 0 |
| Syphilis, - - - - - | 4 | 4 | 0 |
| Ulcers, - - - - - - | 8 | 1 | 1 |
| Ununited fracture, - - - | 1† | 0 | 0 |
| Wounds 3, viz.: | | | |
| Incised, - - - - - - | 1 | 0 | 0 |
| Lacerated, - - - - - | 0 | 0 | 1‡ |
| Penetrating, - - - - - | 0 | 0 | 1 |
| | 47 | 8 | 5 |

*Incised wound of the wrist.—Division of the radial and ulnar arteries and of the median nerve.*—A boy aged 14 was admitted March 19th, 1849, with an incised wound of the left wrist, by which the radial and ulnar arteries, the median nerve, and the superficial flexor tendons of the fore arm were divided. The arteries had been secured by ligatures at both ends before his admission. There was perfect loss of sensation in the hand, except in the thumb and little finger, but the temperature was very slightly changed. The wound was closed by two stitches and adhesive plaster, dressed with dry lint and confined on a straight splint. In three days there was some sensibility in the palm of the hand, in seven days the sensation in the middle finger was natural, and in three days more the fore and ring fingers had recovered their ordinary power of feeling. The ligatures were all separated by the tenth day, and the wound had almost entirely healed by the first intention. He was discharged well April 14th.

He had not recovered the power of perfectly flexing the fingers, but, no doubt, as the union of the divided tendons becomes more firm, their usual strength will be restored.

*Dislocation backwards and a little outwards of both bones of the fore arm without fracture.*—This accident, so rare in the adult, unconnected with fracture either of the internal condyle of

* Died of tubercular meningitis.

† Cured by amputation.

‡ Died of erysipelas of the head.

the humerus or of the coronoid process of the ulna, happened March 19th, 1849, to a very muscular man, a drayman, who had fallen forwards from a bale of cotton and had struck the ulnar edge of the fore arm against the curbstone. He was admitted into the hospital about half an hour after the accident. The nature of the injury was very evident. The hand was pronated, the fore-arm partly flexed on the arm. The olecranon was one inch and a half above its natural position, the internal condyle very prominent, and the head of the radius could be felt and seen rotating a little above the site of the external condyle, which could not be felt.

Reduction was effected by drawing upon and bending the fore arm, with the knee in the bend of the elbow. The bones returned to their places with a loud snap.

The arm was laid on an angular splint, and cold applied. At the end of a week, passive motion was commenced, and at the end of three weeks more he was discharged with all the motions of the joint perfect.

*Wound of the intestine—death.*—A sailor, aged 28, was admitted the night of March 20th, 1849, with a stab of the right iliac region, immediately above the anterior superior spinous process. About four inches of the large intestine, apparently the head of the colon, were protruded through the wound, and in the protruded portion there was a wound about three-fourths of an inch in length, through which fæcal matter was escaping. The injury had been received about an hour before admission, and the patient was so intoxicated as to be unable to give an account of how it happened. His pulse was frequent and full, and his skin warm.

The wound in the intestine, which was longitudinal, with the mucous membrane considerably everted, was closed by the continued suture, one end of which was allowed to depend externally; and the gut, after slight dilatation of the parietal wound, was returned into the abdomen. The external wound was then closed by two stitches and adhesive plaster, and dressed with dry lint.

The patient was ordered fifty drops of laudanum, to be repeated in two hours if he did not sleep. Barley water for diet and perfect rest.

In the morning he was found very restless, with great trembling of the hands, vomiting and other symptoms of delirium tremens.

Ordered ℥j. brandy, and fifty drops laudanum, to be followed by twenty-five drops laudanum every two hours if not more composed. Trickling from the wound of a light coloured liquid. Pulse 120, rather full.

21st. Has had scarcely any sleep, still dreading the horrors. Pulse 128, very compressible. Abdomen slightly tympanitic.

22d. Pulse 138, moderately full. Some tympanites and tenderness of the abdomen; vomiting of a yellowish fluid. Ordered warm fomentations to the abdomen, and an enema which brought away a few hardened fæces.

23d. Pulse 138, soft. Has had some sleep. Abdomen softer and less tender, but still vomiting of a dark coloured fluid. Ordered calomel gr. viij., opium gr. iv. Divide into four pills, one every two hours. Also, an enema, which induced a copious fluid stool unmixed with blood. Purulent discharge from the wound.

The following day he appeared better. Pulse 112, moderately strong. Abdomen more soft and less tender. He had during the night two free stools. Secretion of urine about natural. Still some vomiting.

The following day he was much worse. Vomiting of a dark coloured fluid without any particular odour. Skin cool, pulse 128. Delirious at times. He died that night at ten o'clock, five days after having received the wound.

The autopsy was made 16 hours after death.

Rigidity incomplete. There was a wound two inches in length not united, immediately over the anterior superior spinous process of the right ilium. Commencing decomposition in the neighbourhood of the wound.

The thoracic viscera were found healthy.

On opening the abdomen, it was found to contain a considerable quantity of pus, and shreds of lymph. The intestines everywhere adherent to one another, and to the omentum, which was much thickened. The intestine wounded was the colon, about 3 inches from the ilio-cœcal valve.

The colon was slightly contracted at the seat of injury, and joined by rather firm adhesions to the walls of the abdomen, at a point immediately corresponding to the external wound. The wound in the gut was not united, the mucous membrane everted, and the stitches of the suture entirely separated from one side, but still connected to the other. The mucous membrane in the vicinity of the wound was not inflamed. No fæcal matter could be found in the cavity of the peritoneum, nor was there any smell or other sign of fæcal effusion having caused the fatal inflammation. Kidneys very vascular, and some appearances of extravasated blood in the secreting portion.

*Death from convulsions caused by the irritation of Calculus in the Bladder.*

Francis McFadden, aged 12, was admitted into the Hospital with symptoms of stone in the bladder.

Twelve months before, a stone was removed by lithontripsy, and in the early part of last summer a new formation was removed by the same process. After each operation his health improved very much, but in the last few months the symptoms reappeared with greater severity. About three weeks before admission he was carefully sounded, but no stone detected. Dr. Peace passed a sound into his bladder, and immediately thought he felt a stone, but fearful of increasing the vesical irritation, withdrew the instrument, and ordered him put to bed and anodynes administered.

During the afternoon he suffered intensely with vesicle tenesmus and dysury, and about 10 at night was seized with convulsions which resisted all the remedies employed. In the morning he partly recovered his senses, but was in so exhausted a state that he died a few hours after, a little over 24 hours after he was admitted into the house.

It was afterwards stated by his mother that he had been in such constant pain for the few days preceding his death, that he had been unable to sleep or even to take his food.

The urinary organs were examined 16 hours after death. The kidneys were very pale, lobulated and very much enlarged. The right kidney measured five inches in length, by two and a half in width. The left rather less. The pelves and infundibula very much dilated, and the ureters so much enlarged as to resemble the small intestines. In one of the dilated infundibula of the left kidney, was found a calculus the size of a large pea; the other infundibula contained a small quantity of pus.

On opening the bladder it was found to contain a stone 1½ inch in length, weighing 246 grs. The mucous membrance was injected, and covered with a thick mucus, and it, as well as the muscular coat, was very much thickened. There were no pouches in the bladder; the prostate gland appeared healthy.

The calculus found in the kidney was the triple phosphate. That in the bladder was the triple phosphate with a thick loose coating of phosphate of lime. The whole formed on a nucleus, consisting of a fragment distinctly recognisable.

Permission to examine the head was refused. Most probably no organic change would have been found, as it is reasonable to suppose that the convulsions depended upon cerebral irritation, propagated to the brain from the bladder and kidneys.

Spencer Sergeant,
Resident Physician.

*Pennsylvania Hospital, April 15th,* 1849.

